# Segmental Clavicle Fracture in a Polytraumatized Patient: Case Report

**DOI:** 10.1055/s-0041-1724086

**Published:** 2021-03-31

**Authors:** Carlos A. Sánchez, Pablo J. Coronel, Luisa F. García, Juan S. Afanador, Raúl Gonzalez

**Affiliations:** 1 Ortopedia e Traumatologia, Pontificia Universidad Javeriana, Bogotá, Colômbia; 2 Departamento de Ortopedia e Traumatologia, Hospital Universitario de la Samaritana, Bogotá, Colômbia; 3 Universidad de la Sabana, Chía, Colômbia

**Keywords:** clavicle, fractures fixation, orthopedic surgery

## Abstract

Clavicle fracture represents 5% of the fractures in adults. However, segmental clavicle fractures are infrequent and have been understudied in the current literature. Cases have been reported showing adequate results with both surgical and conservative management, and yet, it has not been possible to reach a consensus regarding diagnosis or management of such condition.

A patient with a middle and lateral segmental clavicle fracture is reported, after presenting multiple trauma in a road traffic accident, also suffering trauma to the right hemi body, multiple rib segmental fractures and hemothorax. After stabilization, he was taken to surgery for open reduction and internal fixation of the clavicle using a double plate technique, as it has been rarely described in the literature. The functional result was shown to be adequate and satisfactory in the postoperative follow-up.

Despite the limited evidence available on the management of this type of pathology, surgical management is a valid option given the risk of non-union. The foregoing is concluded by the potential harm in patient functionality.

## Introduction

Clavicle fractures are quite common, accounting for up to 5% of bone injuries in adults and 44% of those that occur in the shoulder girdle. 1 They appear more frequently in the middle third (69%), followed by the distal third (28%), the proximal third (3%) 2 and the segmental pattern (0.8%). 3 They have two peaks: the first, in young adults, predominantly men, secondary to direct injuries when exercising and to high-energy trauma; the second, in older women with osteoporosis. 2 4

Segmental clavicle fractures are unusual, but they occur in high-energy trauma associated with other injuries, such as rib or scapula fractures. 3 4 The literature is scarce, and it is mainly based on case reports. There is no consensus on pathophysiology or management. 5

The present study presents the case of a patient with a segmental clavicle fracture, secondary to high-energy trauma and associated injuries, along with its management and evolution.

## Case Report

**Fig. 1:**
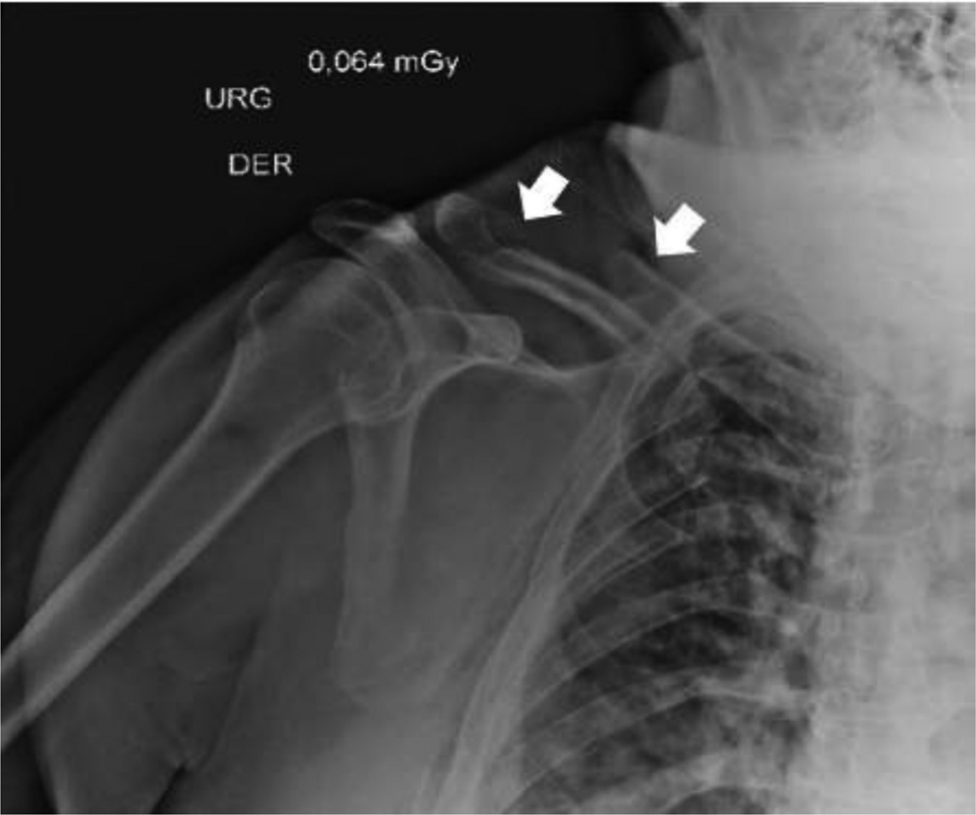
Robinson type 2B2 segmental fracture of the right clavicle. Arrows pointing at both fractures.

A 57-year-old male patient was admitted to the emergency department after polytrauma in a road traffic accident. He suffered direct trauma to the right hemithorax when ejected from the car. There was no evidence of traumatic brain injury. On admission, multiple fractures of the right rib cage, and segmental fractures of the right clavicle (middle third and distal Robinson type 2B2 ( Fig. 1 )) and of the hemothorax were documented.

**Fig. 2:**
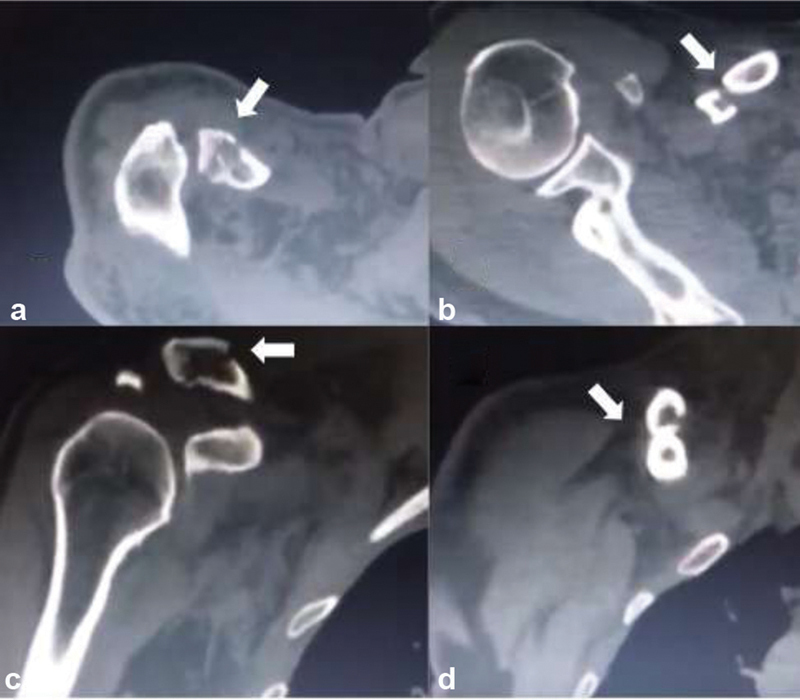
Computed tomography scan of Robinson type 2B2 segmental fracture (a) and (c) Axial and coronal views, lateral fracture (arrow). (b) and (d) Axial and coronal views, middle fracture (arrow).

**Fig. 3:**
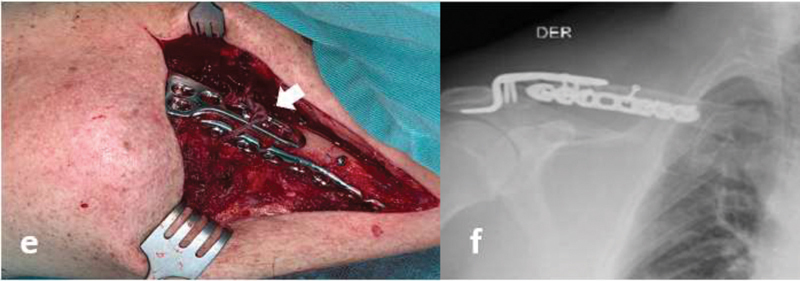
(e) Surgical image, double plate osteosynthesis (anterior and hook plates) and augmentation with FiberTape (arrow). (f) Postoperative X Ray.

**Fig. 4:**
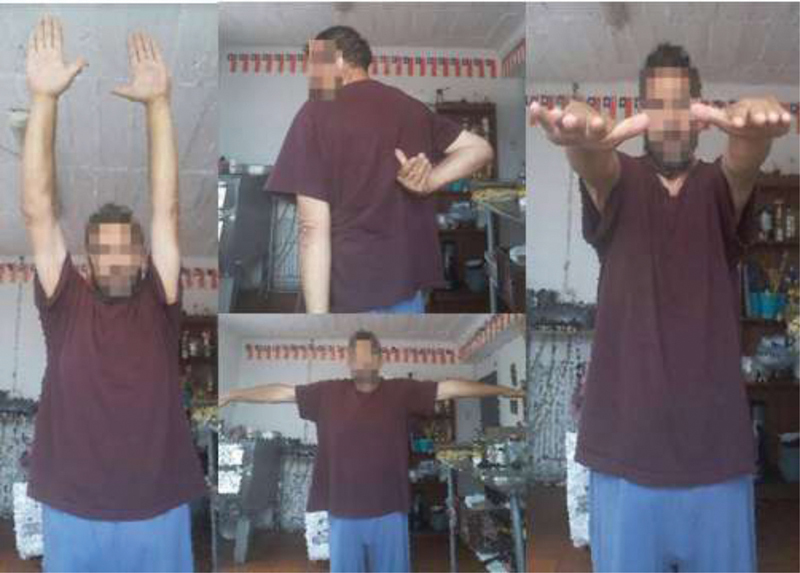
Clinical results after 1 year of surgery.

During the consultation, vascular and nervous lesions were ruled out. The clavicle fracture was better characterized with the use of a computerized axial tomography (CAT) scan ( Fig. 2 ). After controlling for comorbidities, the patient was taken to surgical management. Through a sufficient superior incision and previous plane dissection, the diaphyseal fracture was identified, reduced, and stabilized with a 3.5 cortical lag screw. Then, extending the incision laterally, the second fracture as well as the acromion were exposed, and we identified a small segment that could not be reduced directly, so a 3.5 hook plate was used for indirect reduction. An anterior 3.5 locking compression plate (LCP) plate was used for increasing stability of the construct. Finally, despite using a hook plate and considering the double fracture pattern, augmentation using FiberTape (Arthrex, Naples, FL, USA) around the coracoid process was used to increase lateral stability in the acromioclavicular joint ( Fig. 3 ). The patient presented an adequate evolution after 1 year of follow-up with complete recovery of the range of movement of the right shoulder ( Fig. 4 ).

## Discussion

Segmental clavicle fractures have an incidence of 0.8%, as reported by Throckmorton and Kuhn in 2007. 1 6 7 8 9 They are more frequently observed in men under 60 years old and are associated with high-energy trauma. 6 10 11 12 13 14 On the other hand, they also occur in women over 60 years of age and may be associated with lower energy trauma. 4 7

There is no consensus regarding the trauma mechanism of a segmental clavicle fracture, but it seems to be connected to high energy trauma with associated injuries or even two successive traumas. 1 2 4 6 7 8 10 11 12 13 14 15

These types of fractures are usually observed in radiographs using the usual shoulder and clavicle projections. 1 2 3 4 7 8 11 12 13 14 16 17 It has also been reported that the diagnosis may be belated, especially in polytrauma patients due to the non-identification of one of the two fracture lines on conventional radiography, thus requiring a CAT scan. 4 5 6 7 10 11 15

The results in the literature are diverse, and no consensus has been reached regarding the best management for this type of fracture. 1 4 5 7 8 10 13 15 17 The majority of the cases described in the literature are based on case reports and series, and although studies reinforce non-consensus regarding management, a greater number of case reports opts for surgical management arguing the risk of non-union. 1 2 3 4 5 7 8 10 11 17

In cases describing surgical management, there does not seem to be any trend regarding the best choice for osteosynthesis. The use of locked plates has been the most reported, as well as stabilization with Kirschner wires using tension band wiring procedure and even experimental methods. 1 3 6 8 10 11 12 13 14 16 17 The use of a double plate has been described in some cases with an adequate result, even when performed in two stages. 2 5 7

It seems that the best available evidence appears in the study by Malkoc et al., 3 comparing the results of two groups managed differently, with similar consolidation and functionality, but with better pain control in the group undergoing surgery.

In general, the studies report adequate results, regardless of the management option, except for some cases that require a change from orthopedic to surgical management. 7

This type of fracture is infrequent, requiring a suitable radiological evaluation, especially in polytrauma patients. The correct diagnosis will provide the best management for each case, with the caveat that there is no evidence of the superiority of either orthopedic or surgical management.
