# Supplementary material for: Segmental Clavicle Fracture in a Polytraumatized Patient: Case Report
Source: Rev Bras Ortop (Sao Paulo). 2021 Mar 31;59(1):e139–42. doi: 10.1055/s-0041-1724086 (PMC10957284; doi:10.1055/s-0041-1724086)
Supplement: Related texts

# Research article: Fratura segmentar da clavícula em paciente politraumatizado: Relato de caso

**Keywords:** clavícula, fixação de fraturas, cirurgia ortopédica

## Abstract

A fratura de clavícula representa 5% das fraturas em adultos. No entanto, as fraturas segmentares da clavícula são infrequentes e pouco estudadas na literatura atual. Há relatos de casos com bons resultados após o tratamento cirúrgico ou conservador, mas não existe consenso quanto ao diagnóstico ou manejo dessas lesões.

Relata-se um paciente com fratura segmentar média e lateral da clavícula após politraumatismo em acidente de trânsito. O paciente também apresentava traumatismo em hemicorpo direito, múltiplas fraturas segmentares da costela e do hemotórax. Após estabilização, ele foi encaminhado à cirurgia para redução aberta e fixação interna da clavícula pela técnica de placa dupla, raramente descrita na literatura. O resultado funcional foi considerado satisfatório e adequado no acompanhamento pós-operatório.

Apesar das poucas evidências sobre o manejo desse tipo de patologia, o tratamento cirúrgico é uma opção válida devido ao risco de não união, apesar da possível diminuição da funcionalidade do paciente.

## Introdução

As fraturas da clavícula são bastante comuns, correspondendo a até 5% das lesões ósseas em adultos e 44% daquelas que ocorrem na cintura escapular. 1 Aparentemente, são mais frequentes no terço médio (69%), seguido pelo terço distal (28%), terço proximal (3%) 2 e padrão segmentar (0,8%). 3 Apresentam dois picos: o primeiro, em adultos jovens, predominantemente no sexo masculino, decorrente de lesões diretas durante exercícios e traumas de alta energia; o segundo, em pacientes do sexo feminino, mais velhas e com osteoporose. 2 4

As fraturas segmentares da clavícula são incomuns, mas ocorrem em traumas de alta energia associados a outras lesões, como fraturas de costela ou escápula. 3 4 A literatura é escassa e baseada principalmente em relatos de casos. Não há consenso sobre sua fisiopatologia ou tratamento. 5

Descrevemos no presente trabalho o caso de um paciente com fratura segmentar da clavícula, secundária a trauma de alta energia e lesões associadas, com discussão de seu tratamento e evolução.

## Relato do caso

Um paciente do sexo masculino, de 57 anos, deu entrada no pronto-socorro por politraumatismo em acidente de trânsito. O paciente sofreu trauma direto no hemitórax direito ao ser ejetado do veículo. Não havia evidências de lesão cerebral traumática. À internação, múltiplas fraturas da caixa torácica direita, fratura segmentar da clavícula direita (terço médio e distal, classificada como tipo 2B2 de Robinson [ Fig. 1 ]) e do hemotórax foram documentadas.

**Fig. 1:** Fratura segmentar do tipo 2B2 de Robinson da clavícula direita. As setas indicam as duas fraturas.

Durante o atendimento, a presença de lesões vasculares e nervosas foi descartada. A fratura da clavícula foi caracterizada com o uso de tomografia computadorizada (TC) ( Fig. 2 ). Após o controle de comorbidades, o paciente foi encaminhado para tratamento cirúrgico. Por meio de incisão superior suficiente e dissecção por planos, a fratura diafisária foi identificada, reduzida e estabilizada com parafuso cortical de tração ( *lag* ) de 3,5 mm. Em seguida, após a extensão lateral da incisão, a segunda fratura e o acrômio foram expostos, e houve identificação de um pequeno segmento não passível de redução direta. Esse segmento foi submetido à redução indireta com placa de gancho de 3,5 mm. Uma placa de compressão anterior (LCP, na sigla em inglês) de 3,5 mm foi usada para aumentar a estabilidade do construto. Por fim, apesar da utilização de placa de gancho e considerando o padrão de dupla fratura, usamos FiberTape (Arthrex, Naples, FL, EUA) ao redor do processo coracoide para aumentar a estabilidade lateral da articulação acromioclavicular ( Fig. 3 ). O paciente apresentou evolução adequada após 1 ano de acompanhamento, com recuperação completa da amplitude de movimento do ombro direito ( Fig. 4 ).

**Fig. 2:** Tomografia computadorizada de fratura segmentar de tipo 2B2 de Robinson. (a) e (c), Projeções axial e coronal, fratura lateral (seta). (b) e (d) Projeções axial e coronal, fratura medial (seta).

**Fig. 3:** (e) Imagem cirúrgica, osteossíntese de placa dupla (placa anterior e placa de gancho) e aumento com FiberTape (seta). (f) Radiografia pós-operatória.

**Fig. 4:** Resultados clínicos 1 ano após a cirurgia.

## Discussão

A incidência de fraturas segmentares da clavícula é de 0,8% segundo Throckmorton e Kuhn, em 2007. 1 6 7 8 9 Esse tipo de fratura é mais comum em homens com menos de 60 anos e está associada a traumas de alta energia. 6 10 11 12 13 14 Por outro lado, também ocorre em mulheres com mais de 60 anos e pode estar associada a traumas de menor energia. 4 7

Não há consenso quanto ao mecanismo de trauma de uma fratura segmentar da clavícula. Esse tipo de fratura, porém, parece ligado a traumatismos de alta energia com lesões associadas ou mesmo dois traumatismos sucessivos. 1 2 4 6 7 8 10 11 12 13 14 15

Esses tipos de fraturas são geralmente observados em radiografias nas projeções usuais de ombro e clavícula. 1 2 3 4 7 8 11 12 13 14 16 17 Há também relatos de diagnóstico tardio, principalmente em pacientes politraumatizados, devido à não identificação de uma das duas linhas de fratura em radiografias convencionais, com necessidade de TC. 4 5 6 7 10 11 15

A literatura relata desfechos diversos, sem consenso quanto ao melhor tratamento para esse tipo de fratura. 1 4 5 7 8 10 13 15 17 A maior parte da literatura é composta por relatos e séries de casos e, embora os estudos reforcem a ausência de consenso quanto ao tratamento, um número maior de relatos de casos opta por cirurgias devido ao risco de não união. 1 2 3 4 5 7 8 10 11 17

Nos casos que descrevem o tratamento cirúrgico, não parece haver tendência quanto à melhor escolha da osteossíntese. O uso de placas de bloqueio é o mais relatado, bem como a estabilização com fios de Kirschner utilizando procedimentos com banda de tensão e até métodos experimentais. 1 3 6 8 10 11 12 13 14 16 17 O uso de placa dupla foi descrito em alguns casos com bons resultados, mesmo quando realizado em duas etapas. 2 5 7

Aparentemente, as melhores evidências são do estudo de Malkoc et al., 3 no qual foram comparados os resultados de dois grupos submetidos a tratamentos diferentes. A consolidação e a funcionalidade foram semelhantes, mas o controle da dor foi melhor no grupo operado.

De modo geral, os estudos relatam resultados adequados, independentemente da opção terapêutica, exceto em alguns casos com necessidade de mudança de tratamento ortopédico para cirúrgico. 7

Esse tipo de fratura é incomum e requer avaliação radiológica adequada, principalmente em pacientes politraumatizados. O diagnóstico correto determina o melhor tratamento para cada caso, com a ressalva de que não há evidências da superioridade de abordagens ortopédicas ou cirúrgicas.
